# Building Large Collections of Chinese and English Medical Terms from Semi-Structured and Encyclopedia Websites

**DOI:** 10.1371/journal.pone.0067526

**Published:** 2013-07-09

**Authors:** Yan Xu, Yining Wang, Jian-Tao Sun, Jianwen Zhang, Junichi Tsujii, Eric Chang

**Affiliations:** 1 State Key Laboratory of Software Development Environment, Key Laboratory of Biomechanics and Mechanobiology of Ministry of Education, Beihang University, Beijing, China; 2 Microsoft Research Asia, Beijing, China; 3 Institute for Interdisciplinary Information Sciences, Tsinghua University, Beijing, China; University of Warwick, United Kingdom

## Abstract

To build large collections of medical terms from semi-structured information sources (e.g. tables, lists, etc.) and encyclopedia sites on the web. The terms are classified into the three semantic categories, Medical Problems, Medications, and Medical Tests, which were used in i2b2 challenge tasks. We developed two systems, one for Chinese and another for English terms. The two systems share the same methodology and use the same software with minimum language dependent parts. We produced large collections of terms by exploiting billions of semi-structured information sources and encyclopedia sites on the Web. The standard performance metric of recall (R) is extended to three different types of Recall to take the surface variability of terms into consideration. They are Surface Recall (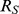), Object Recall (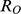), and Surface Head recall (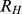). We use two test sets for Chinese. For English, we use a collection of terms in the 2010 i2b2 text. Two collections of terms, one for English and the other for Chinese, have been created. The terms in these collections are classified as either of Medical Problems, Medications, or Medical Tests in the i2b2 challenge tasks. The English collection contains 49,249 (Problems), 89,591 (Medications) and 25,107 (Tests) terms, while the Chinese one contains 66,780 (Problems), 101,025 (Medications), and 15,032 (Tests) terms. The proposed method of constructing a large collection of medical terms is both efficient and effective, and, most of all, independent of language. The collections will be made publicly available.

## Introduction

Building large collections of medical terms [1]–[4] is of great importance as it is the first essential step for medical text processing (such as named-entity recognition, relation extraction, etc). While sophisticated algorithms have been developed for medical record processing, a lack of a large collection of terms that occur in actual medical reports is one of the major obstacles for these algorithms being deployed in the real world. There are collections of terms for English such as Unified Medical Language System (UMLS) [5], Medical Subject Heading (MeSH) [6], Systematized Nomenclature of Medicine-Clinical Terms (SNOMED) [7], and DrugBank [8], which are rich in standardized and formal terms, but they tend not to cover terms in actual medical records. As for Chinese [9]–[12], even such collections of formal medical terms are still poor in content, compared with those for English.

In this paper, we describe how we have built large collections of terms that can be used as sharable resources by the community of medical record processing. Since the methods used are mostly language-independent, and require minimum manual intervention, they can be applied to gathering terms for languages other than Chinese and English. Thus, our work can also contribute to medical record processing in local languages, which has become increasingly important.

The automatic creation of term collections and the building of thesauri based on them have been studied in the general domain. The extensive use of Web resources [13] has characterized their attempts. However, due to the peculiar characteristics of medical terms and medical records as text, their straightforward application is not effective. To our knowledge, our method is the first attempt to create medical term collections in this direction. Our methods need a set of seed terms to start with. We use BaiduBaike [14], which is like Wikipedia [15] but lager than Chinese Wikipedia, to create the initial seeds for Chinese, while we use terms in the i2b2 corpus [16] as seeds for English. The first challenge is to gather a large collection of candidates from the Web by using these seed terms. The second challenge is to filter out noise (non-terms) from the candidate list. Similar to methods in the general domain, we use a search engine (i.e. Bing Search) for the first challenge. We submitted seeds to Bing Search and downloaded the webpages returned by Bing. Instead of processing text to extract candidates, we used “parallel” structures such as tables and lists in web pages to extract candidate terms. This is because web pages on medical information often use structures such as tables and lists to present information on drugs, diseases, etc. These structures have proven to be very useful for identifying terms that rarely appear on the Web as a whole.

The second challenge, filtering out noise from candidate lists, is harder than the first. In our system, we've combined three different techniques. The first method is snippet analyses that use a naïve Bayes classifier to decide whether a candidate is a valid medical term of a given semantic category or not. The three classifiers, which correspond to the three semantic categories, use a set of snippet contexts in which a candidate appears. The second method is based on an assessment of the appropriateness of “parallel” structures. If a candidate appears in a parallel structure that contains many known terms of the same category, the candidate can be judged as a valid term of the category. The third one is based on the surface form of a candidate. Because of terminological conventions in the medical domain, terms tend to share common morphological characteristics (e.g. affixes in English, a set of specific characters in Chinese). By combining these three methods, we've developed a system that is both efficient and effective at gathering medical terms.

The contributions of this paper are four-fold. First, it is the first attempt to gather medical terms using resources on the Web. The second contribution is a novel and generic method of expanding a set of seed terms to obtain a much larger collection of terms in the same semantic categories. The method is generic in the sense that it can be applied to languages other than English and Chinese. The whole system is novel in the sense that it uses the characteristics of medical terms and their occurrences on Web pages extensively by modifying term gathering methods in the general domain. The third contribution is the evaluation of the quality of term collections by using actual medical records (the i2b2 corpus, Chinese translation of the i2b2 terms and Chinese medical records manually annotated by domain specialists). Finally, we will make the collections publicly available, which will contribute to the research community of medical record processing in English and Chinese.

This paper describes the details of seed creation, the three methods of noise filtering, and the experiments for a quality evaluation of the term collections. The experiments show that the system is both efficient and effective at collecting medical terms from the Web.

## Related Work

Our method focuses on exploiting parallel structures from the Internet. Parallel structures on a webpage include lists, tables, and navigation bars. These parallel structures contain many more rare-occurring entities than they do text on the Web. There have been extensive studies on exploiting the tables. Yoshida et al [17] extracted ontologies or simple semantic classes from HTML tables. They first assumed that there were nine types of tables according to the configuration of rows and columns. Then they used the EM algorithm, which performs identification of table types and the formation of semantic classes simultaneously. Cafarella et al [18] built a search engine based on a corpus of 14.1 billion HTML tables. Three novel applications, schema auto-complete (helping a database designer to choose schema elements), attribute synonym finding (computing attribute synonym pairs for schema matching), and joint-graph traversal (using joint links to navigate between extracted schemas), were developed based on the table search engine. Limaye et al [19] proposed a graphical model to annotate entities from table cells, types from table columns, and relationships that pairs of table columns are intended to express. Yakout et al [20] used a collection of tables for augmenting entity information by adding the attribute names and their values to an entity. While relevant to our work, these studies focused on ontology acquisition and entity information enrichment. Our objective in this paper is different. We use “parallel” structures, including tables, to gather as many names of entities as possible. In particular, most of the specialized terms do not occur so frequently. Exploiting the “parallel” structures that enumerate rare-occurring terms constitutes the key to our method. Furthermore, our method combines the analysis of “parallel” structures with the analysis of textual information (e.g. snippets). These two analyses have been studied separately.

Text analysis for ontology building has been studied extensively from the perspective of Distributional Semantics (DS). DS uses co-occurrence information to calculate the similarity among terms. Curran [21] evaluated existing and new similarity metrics and then proposed a method, called an approximation algorithm, to improve the time complexity and execution time of similarity calculation. Curran [22] proposed k-nearest-neighbor matching for thesaurus extraction, which calculates the pairwise similarity of target terms. In Chinese, Tseng [23] proposed a classifier to assign semantic categories to unknown Chinese words. Morphological similarity among words is used. Kwong [24] assigned an existing semantic classificatory structure to new words. The cosine function for similarity was used. Like table analyses, textual analyses based on DS have focused more on thesaurus or ontology construction than gathering a large number of terms of given semantic classes, which is the objective of this paper. Frantzi et al [25] proposed the C-value and NC-value method, which integrates linguistic and statistical information to extract multi-word terms from English corpora. Although our proposed method also uses contextual information as well as statistical models to perform terminology extraction tasks, the objective of the C-value method is significantly different from ours: rather than focusing on accurately extracting all terms from a specific document, we are more interested in creating a large collection of terms from the Internet for practical uses, which involves finding documents and webpages with rich medical terms, a task C-value does not attempt to solve. On the other hand, missing terms in a single document is allowable for our objective, as long as they can be extracted from other resources.

In this paper, we propose a baseline method of exploiting the parallel structures in Web to build English and Chinese collections of medical terms. On the premise of guaranteeing high precision of dictionary, our method produces a large collection of terms for given semantic classes with a reasonable time complexity.

## Methods

**Figure 1:**
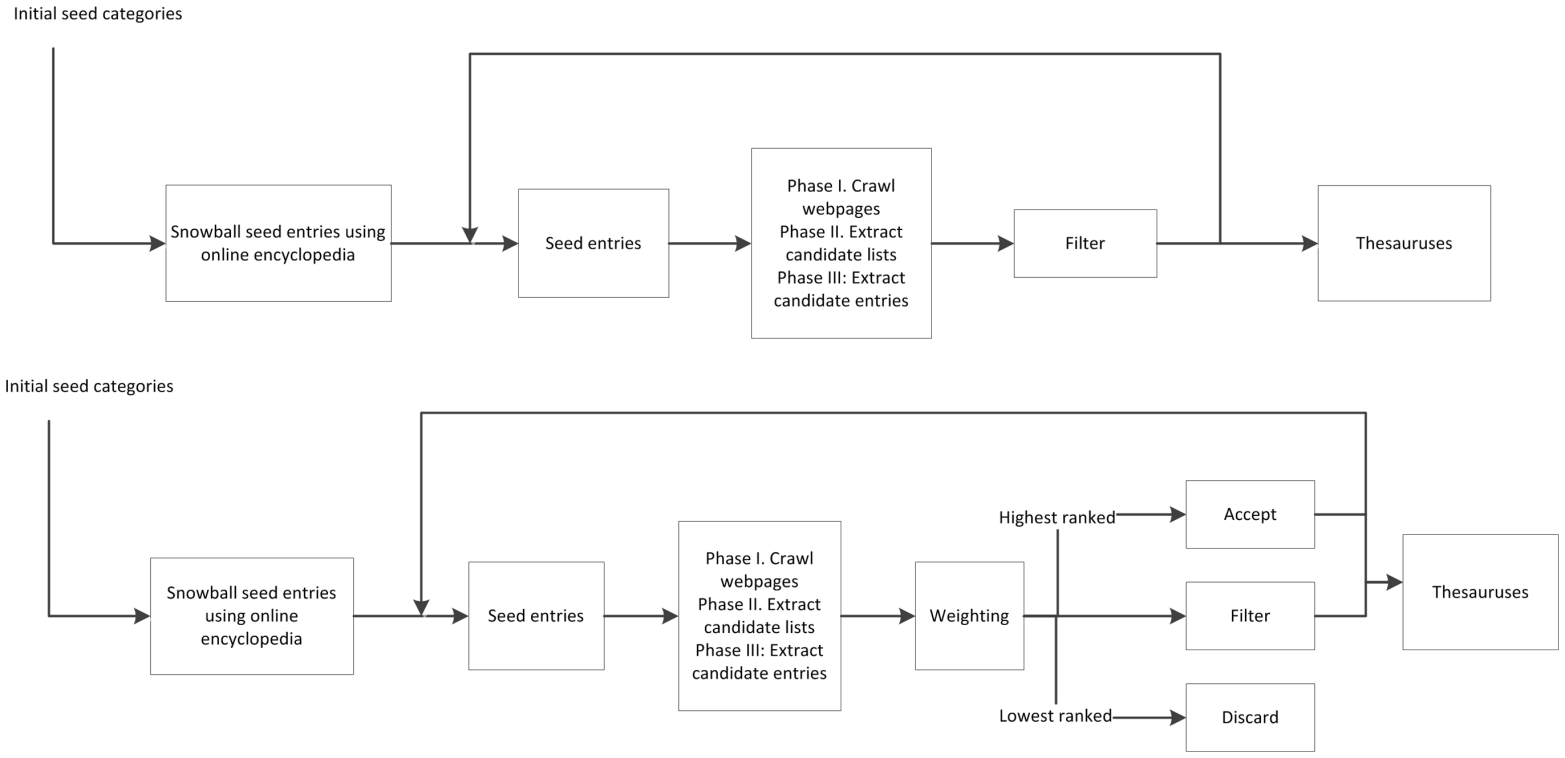
The upper graph depicts the baseline algorithm while the lower graph depicts the improved algorithm.

Two systems were constructed. The difference between the two lies in the filtering phase. The first system is solely based on snippet analysis for filtering out noise, while the second one combines the three methods. The two systems were evaluated using the same set of seeds and evaluation sets. Fig. 1 shows the architecture of the two systems.

### Creation of Seed Sets

The i2b2 challenge tasks in 2010 provided 826 discharge summaries with 72,846 terms (Medical Problems, Treatments, and Tests). Since these terms are from actual medical records, we use the terms in 176 summaries as seeds for English in the Problem and Test sets. Since Treatment consists of heterogeneous subclasses, we only dealt with Medication. The seeds for Medication were derived from the medication dictionary of SNOMED, independently of the i2b2 corpus. The rest of the discharge summaries were used as the test corpus. Duplicate terms and modifiers such as “his” and “her” in i2b2 annotations were removed. The set of seeds thus obtained contains 20,380 distinct terms.

**Figure 2:**
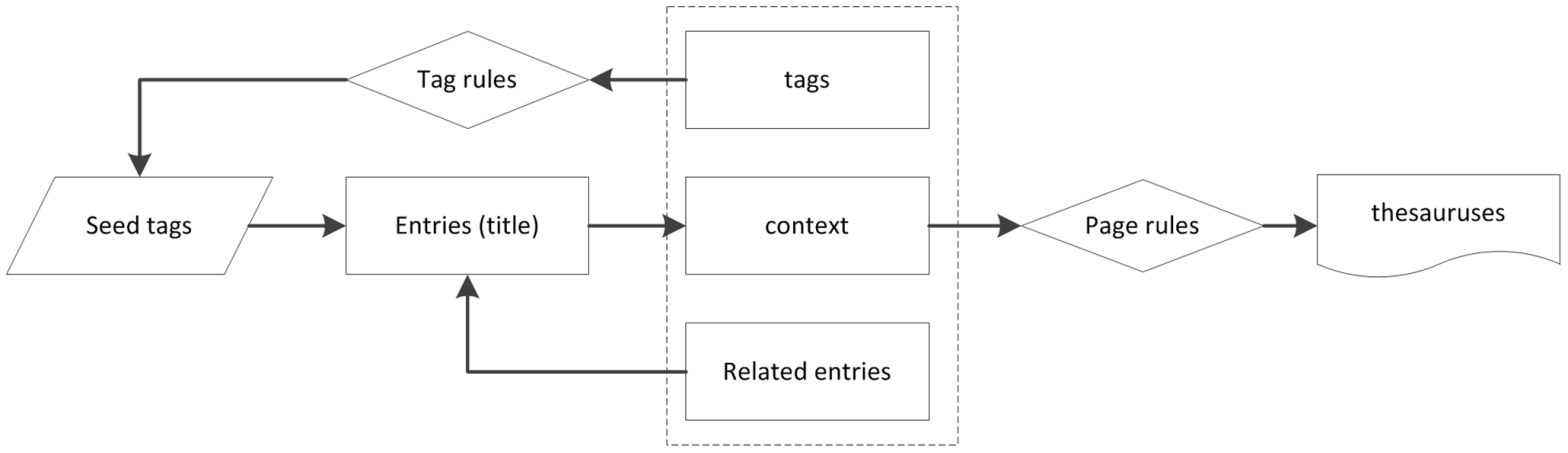
The diagram block of Chinese seed thesauruses extraction.

For Chinese, since we did not have suitable resources for seeds, we used BaiduBaike, the largest online encyclopedia in Chinese (http://baike.baidu.com/), to create a set of seeds. The diagram block is shown in Figure 2. An entry in BaiduBaike consists of four parts: 1) Title: the name of the entry; 2) Content: description or explanation of the entry; 3) Tags: categories that the entry belongs to; 4) Related entries: entries related to the entry. The entry of *leukemia*, for example, has *cancer* and *hematopoietic system diseases* as Tags and *gastric cancer* and *anemia* as related entries.

We first choose a set of seed tags (ST) for the three categories (such as 药物 (drug) and 医药 (drug)) and gather a set of entries that have the tags. The set of entries are further enlarged by adding the entries of related terms and by adding other tags as seed tags that appear in the added entries. Since the terms gathered at this stage are used as the seeds for the next stage, precision is the main concern. The coverage (i.e. Recall) of a collection is taken care of by the next stage. The number of terms that are less representative, and thus not registered as entries in a Web encyclopedia, is much larger than those enlisted as entries. The next stage that uses these seeds is responsible for gathering such terms.

In order to maintain the high precision of collected terms, we use a set of strict rules for checking descriptions of entries to choose only entries that are certain about the terms in the three categories. We use a set of keywords manually chosen as page rules. For instance, for Drug, we adopt terms as seeds only if their pages in BaiduBaike contain the keyword “不良反应” (adversary/side effects), and otherwise discard the terms as seeds.

As a result of this process, we collected 6,599 terms of Problem, 10,700 terms of Medication and 1,424 terms of Test in Chinese.

### Collecting of Candidates of Terms

The set of seed terms is used to gather a larger set of terms from the Web. A set of term candidates is collected in four phases: 1) Identifying webpages that may contain “parallel” structures of medical terms, 2) Using “parallel” structures to create lists of candidates for a chosen page, 3) Selecting proper candidate lists for the page, and 4) Creating a set of candidate terms that is collected from candidate lists of all the pages. The set of term candidates is passed to the final stage, e.g. the phase of filtering out noise (non-terms) from the set. In the following section, we first describe the four phases in detail, and then the three methods of filtering noise.

### Phase I. Identification of webpages that may contain “parallel” structures in medical terms

A collection of webpages that may contain many terms is created by issuing queries to an existing search engine (e.g. Bing Search). Since queries of single seeds return too many pages, which only contain the single input seeds, we use queries that consist of two terms from the set of seed terms belonging to the same semantic category. This phase identifies 110,000 pages (Problems), 75,000 pages (Medications), 33,000 pages (Tests) for Chinese and 165,000 pages (Problems), 300,000 (Medications), 200,000 (Tests) for English as pages that may contain “parallel” structures of medical terms.

### Phase II. Extraction of parallel structures from crawled webpages

In this paper, we use the term “parallel” structure to denote a list, a table, an index page, etc. in which items of the same type are enumerated. Since a webpage selected by Phase I contains at least two medical terms of the same class, the likelihood that the page contains such enumeration is high.

**Figure 3:**
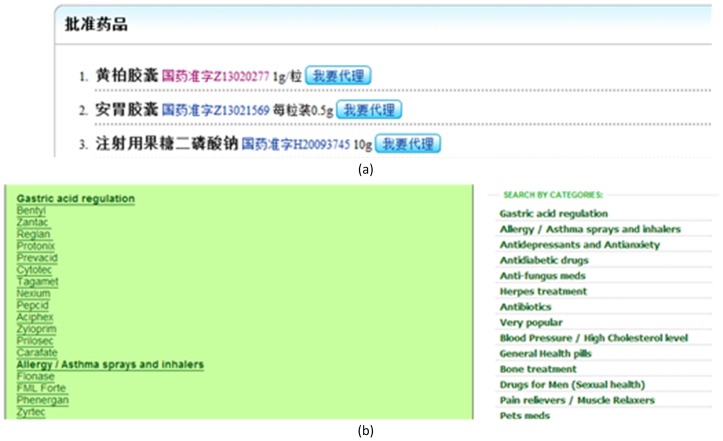
An example of parallel named entities in HTML in Chinese (a) and English (b).

**Figure 4:**
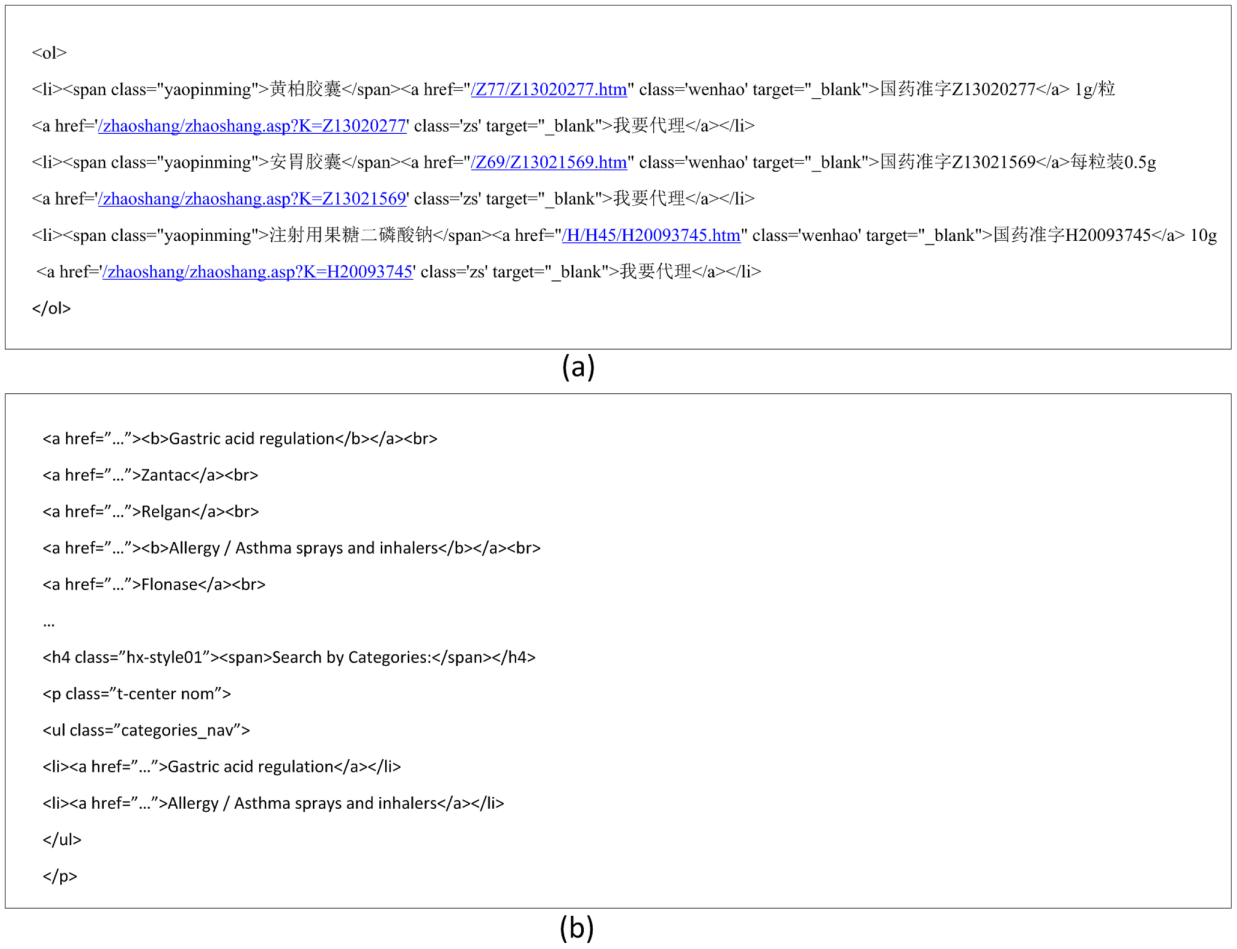
The HTML text of the list in Figure 3 in Chinese (a) and English (b).

We assume that medical terms enumerated in such a parallel structure are located in similar positions of HTML DOM trees (tag trees), and that characters of medical terms in enumeration share the same font types and sizes. As illustrated in Figure 3 and Figure 4, all drug names are bold and in a larger font while all approval numbers are in a smaller font.

We define *tag paths* and *tag-class paths* of nodes in HTML DOM trees as follows:

- Tag path: A *tag path* of a node is the path from the root to the node in the DOM tree (tag tree).
- Tag-class path: A *tag-class path* of a node is its *tag path* with the class of each tag. In case a tag does not have a class specified, we deem the class “empty.”

**Figure 5:**
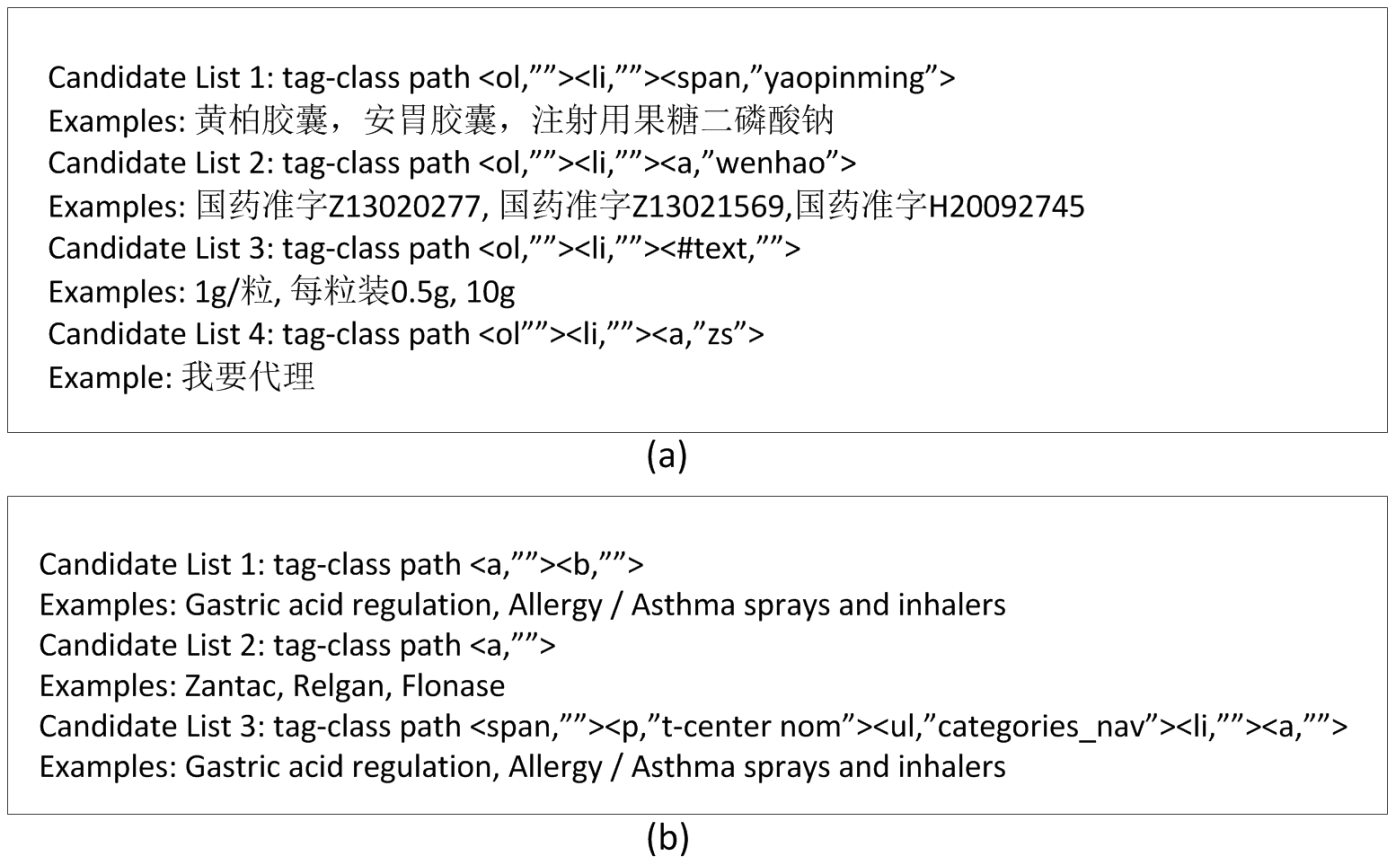
Some of the extracted candidate lists from the webpage in Figure 3 in Chinese (a) and English (b).

When two nodes in the DOM tree have the same tag-class path, they occupy similar positions in a parallel structure and their characters share the same fonts and sizes. Therefore, we collect all text nodes with the same tag-class paths into one candidate list. For instance, in Figure 5, the items are grouped into 4 different candidate lists (for drug names, approval numbers, dosages, and purchase requirements) according to their tag-class paths.

### Phase III: Selection of candidate lists

Candidate lists produced by Phase II are not necessarily lists of medical terms. For instance, in Figure 5, only the first candidate list (drug names) is a list of medical terms. Although they are lists of items of the same types (e.g. dosage information, etc.), their types are of no interest for our current objective.

We only choose lists that contain more than k seed terms (currently, k = 10). Since a list thus selected still has non-terms in it, we clean it up by removing items on the list that are too long or have punctuation symbols in them.

### Phase IV. Creation of a set of term candidates

All lists of candidate terms created from webpages are gathered into a set of candidate terms. For each term, we keep the record of which candidate lists the term comes from. The record is to be used in the final phase of filtering out noise. After this phase, the candidate sets for Chinese contained 1,010,701 items (Medications), 1,306,600 items (Problems), and 500,425 items (Tests), while those for English contained 1,189,214 items (Medications), 600,000 items (Problems), and 600,000 items (Tests).

### Phase V. Filtering out of noises

The sets of candidate terms contain a large number of non-terms. Actually, the number of non-terms in the sets is much larger than that of terms. We developed three methods for removing non-terms from the sets: 1) Snippet Analysis, 2) Selection of Proper “parallel” structures, and 3) Analysis of term forms.

### [1]Snippet analysis

Since the search engine returns a list of webpages with the query word in context (e.g. snippet), one can use the snippet to check the context to judge whether the query word actually is a medical term of a given semantic category. We define a snippet and the context in which a query word (term candidate) appears, as follows:

- *Snippet*: A *snippet* of an entry returned by a search engine consists of the title, URL, and a short context for the page in which the query term appears. When we submit a query, the search engine we use (e.g. Bing) returns many entries with their snippets. We use the top 100 snippets.
- Context pattern: A *contextual pattern* of a query term in a snippet is a set of *N*-grams (N<6) that follow or precede the query term in context. In order to avoid difficulties in detecting the boundaries of a term, we only use the contexts in which the query word appear either in the very beginning of the context or in the end of the context.

Some contexts (e.g. N-grams) are effective for identification of a term in a given class, while others are too general to be useful. Effective N-grams are those that appear frequently in the context of terms of a given class but seldom appear in the context of terms of other classes. Since we have distinct sets of seed terms of the three classes (Medications, Problems, and Tests), we use these seed terms to select a set of effective N-grams.

For a given N-gram *P*, let *N_p_* be the number of occurrences of *P* on the seed terms of a given semantic class, *O* the number of seed terms of the other two semantic classes, and *N_o_* the number of a seed term of the other two classes for which there is at least one occurrence of *P* in its context. We can define a score for measuring the effectiveness of *P* for a given class as follows:
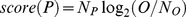
(1)


Note that the score is similar to the TF/IDF, which is effective for choosing informative keywords in Information Retrieval. Examples of N-grams found effective by this score are shown in Table 1.

**Table 1:** Highest-ranked contextual patterns for the three classes (XXX means entity).

Problem	Medication	Test
??(the treatment of XXX)	??? (XXX instruction)	?? (XXX high)
?? (XXX symptom)	??? (XXX side effects)	?? (XXX positive)
??? (XXX, how to deal with it)	???? (XXX instruction)	?? (measure XXX)
????? (XXX, what's wrong with it)	?? (XXX efficacy)	??? (XXX normal value)
??(XXX etiology)	???? (XXX product)	????(XXX clinical significance)

We rank extracted patterns according to their scores and choose the 50 highest ranked patterns for each category. We then train a naïve Bayes classifier for each category. The chosen patterns are used as binary features in the classifiers. For each candidate term, if at least one snippet extracted for the term contains a chosen pattern (N-gram), the corresponding feature is set to 1. For training, all seed terms are used as positive instances, while terms randomly selected from candidate lists whose hit counts are zero (i.e. candidate lists that do not contain any seed terms) are used as negative instances.

We believe that the selection of statistical inference models does not significantly affect the performance of our proposed method. We chose naïve Bayes classifiers because compared to the dimension of the feature space (50), we have many more training instances (>10,000) and hence the posterior probability estimation will be quite accurate. On the other hand, classifiers such as support vector machines are more suitable for fewer training instances with a large dimension of feature vectors.

### [2] Selection of appropriate candidate lists

Snippet analysis is computationally expensive. It is necessary to issue queries for all the term candidates, to process all the snippets (100 at most for each candidate) and then apply the classifiers. Our experiments show that, among the vast number of candidates (e.g. 1,306,600 for medications in Chinese), only a small portion (45,712) are judged as valid terms. Moreover, the snippet analysis is not effective for terms that rarely occur on the Web. For the method to work, a reasonable number of snippets have to be retrieved. As a result, snippet analysis alone does not provide wide coverage and our experiment shows that the Recall is low.

We developed a new method based on the idea of previous table analysis that the same column or row in a table tends to contain items of the same type. We apply the idea to a set of candidate lists extracted by page analysis of **Phase III.** These lists are the results of table analysis and correspond to a row or a column in a table or a list of items in an itemized construction. Therefore, if a list contained more than a certain proportion of seed terms of the same class, we consider the rest of the items on the list as terms of the same type. To reduce the noise we observe in the experiment, we impose a further condition that the average length of items in a list should be within a certain limit. The average length of items in a list is the average number of English words or the average number of Chinese characters for each entry in the list, depending on the language setting.

Let *H_L_* be the number of seed terms on a list (*L*), *N_L_* the number of items in *L*, and 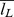 the average length of items on the list. Eq. (2) is used to give a score 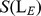 of the quality of the list. Note that we use a logarithm on *N_L_* because the hit-count of a list is more important than its length.
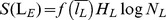
(2)


The *f* function that measures the fitness of an average length is shown in Eq. (3).
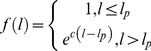
(3)In Eq. (3), we use a fast exponential decay to punish all entries that are too long for a specific domain (long entries may be extracted as fragments of sentences and are less likely to be an in-domain terminology). *l_p_* is a parameter demonstrating the “proper” length of an entry, and differs for different language settings. The parameter *c* is set to the value such that 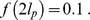 In our algorithm, *l_p_* is set to 5 for Chinese and 2 for English.

After scoring all candidate lists, we rank them according to their 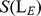 and select the 200 highest ranked lists for each category. Out of 200 lists, we choose those whose number of entities is greater than 100. We add all terms on these lists to the term collections. Table 2 shows that a large number of terms are added at this stage. The step contributes to the coverage for terms that seldom appear on the Web.

**Table 2:** Chosen lists as components of dictionaries.

	English		Chinese	
	# lists	# terms	# lists	# terms
Problem	54	9381	14	21068
Medication	85	58805	37	51427
Test	47	6043	67	9941

### [3] Selection of terms based on their forms and occurrences in lists

[2] uses only a set of lists whose quality is high and contains a large number of terms. However, many lists that are shorter and may contain more heterogeneous pieces of information exist on the web. To exploit such lists effectively, we add a term E whose *S*(*E*) defined in Eq. (4) is higher than a threshold.
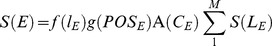
(4)M is the number of the lists where *E* appears. A candidate that appears on lists with reasonably high quality is chosen as a term. However, lists that are shorter or have lower quality in terms of *S*(*L_E_*) tend to contain non-term information as their list elements. The other three functions (f, g, and A) discard such elements (e.g. a kind of phrase that adds extra-information, comments, etc.). They are the length of the list elements (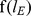), the counts of POS contained in the element (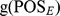), and affixes (A(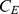)), which are briefly explained in the following section.

#### Entity length (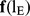)

Length (i.e. number of characters in Chinese and number of words in English) is an important metric indicator. We have observed that in Chinese most terms have 3∼5 Chinese characters, while in English most terms have 1∼3 words. Terms that are longer or shorter should be penalized. We again useEq. (3) to determine whether the length of a term is appropriate and penalize those terms that are too long.

#### POS counts (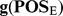)

POS means part-of-speech assigned to words (e.g noun, adjective, verb). We have observed that list elements consist only of words with POSs of adjectives and nouns that are appropriate. An element that contains pronouns (PRP), particles (RP, such as of, up, down, off, etc.) or question words (WP, WRB) are discarded. We use Stanford Taggers for obtaining POSs of words in English and Chinese. The 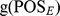 function is defined in Eq. (5).
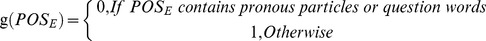
(5)


#### affixes (A(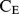))

We have observed that many medical terms have common affixes. For instance, while Chinese medication terms often end with “胶囊” (capsule), disease names end with “炎” (inflammation). Similarly, English medical terms ending with “-scopy” are often a test, like “gastroscopy” and “colonoscopy.”

We first extract affixes from seed terms and rank them in descending order according to their frequency. Note that affixes for Chinese and English terms are morphologically different: for Chinese we use the first and last three characters as affixes of a term, while for English we use the first and last five English letters as affixes. After ranking the affixes, we pick the 20 highest ranked affixes for each category. We then extract binary features for both positive and negative instances for each of these affixes (i.e. whether the word starts/ends with the prefix/suffix). For instance, the word “gastroscopy” has value 1 for both “-scopy” and “-copy” features, but value 0 for the feature “-ia.” After extracting features, we train a naïve Bayes classifier on the training data and use the confidence of an entry as the value of A(C_E_).

### [4] Combined system for filtering non-terms

After [2] and [3], the top 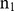 appropriate candidate lists and the top 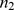 candidate entries are chosen as a member of our expanded dictionary without further filtering. Candidate entries of the lowest score (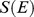) are directly deleted without the snippet processing. The rest is decided via snippet analysis.

## Experiments

In this section, we introduce the reference corpus for evaluation, evaluation methods, and performance for extracted thesauruses.

### Reference corpus for evaluation

Two sets of reference corpora were used for evaluation of Chinese term collections: the i2b2 corpus, and the Chinese labeled corpus.

i2b2 corpus: We randomly selected named entities from the English corpus used in i2b2 open challenge tasks and translated them into Chinese manually. Translation was done without referring to the term collections. We use machine translation systems (e.g. Google translation [26] and Engkoo [27]) to provide the first draft of translation, and then we verify and adjust them manually. For abbreviations, which are common in English medical records and usually cannot be translated by machine translation tools, we refer to Wikipedia disambiguation pages to get full names of the abbreviations. We then translate them in the same way as the other terms. 1,000 name entities are translated for each category.

Chinese labeled corpus: We have a set of discharge summaries in Chinese. The data set is annotated by three clinical doctors and three annotators with a background in computer linguistics. Two of the three doctors are asked to annotate individually, and the other doctor makes the final decision. That is, the third doctor adopts the annotations from the two if their annotations agree with each other. Otherwise, the third doctor decides. For the cases for which the third doctor cannot decide, discussions among the three were organized to reach an agreement. Annotations given by the doctors contain inconsistencies in terms of the boundaries of terms in the text, although the quality of their annotations is high in terms of semantics. To redress the inconsistency, the second round of annotation was performed by three annotators with a background in computer linguistics. The final gold standard of annotated discharge summaries is obtained for 336 discharge summaries in Chinese (A separate paper is in preparation for the details of the annotation). As for English, we use the corpus used in i2b2 open challenge tasks.

### Reference collections of drug names

To test the coverage of collections of drugs produced by our proposed method, we use two reference drug name collections: SNOMED CT and DrugBank. The SNOMED CT is a comprehensive clinical terminology and DrugBank is a database that contains detailed information for 6,712 drug entries. In our experiment, we extracted all drug names from the DrugBank database. Note that the list of drug names we constructed using DrugBank consists of more than 6,712 entries because each entry (i.e. drug) in the database may have many different names, such as generic names, chemical names, brand names, etc. We also extracted all medication terms from SNOMED CT and constructed a list of approximately 10,000 entries, which was also used as seed dictionaries of our proposed method. Please see Table 3 and Table 4 for further details of the two reference collections of drug names.

**Table 3:** Information after expanding thesauruses.

	Categories	No. of terms	No. of removed	Err	Time consumed
**English**					
i2b2 seeds	Problem	6,973	/	/	/
	Medication	9,924	/	/	/
	Test	3,483	/	/	/
Baseline	Problem	39,868	560,132	5.0%	115h
	Medication	30,786	1,158,428	4.0%	90h
	Test	19,064	580,936	3.0%	45h
Weight alg	Problem	49,249	550,751	4.3%	60h
	Medication	89,591	1,099,623	2.3%	55h
	Test	25,107	574,893	2.3%	30h
	Drugbank	34,165			
	Drugbank+SNOMED	41,697			
**Chinese**					
Baidubaike	Problem	6,599	/	/	22h
	Medication	10,700	/	/	24h
	Test	1,424	/	/	10h
Baseline	Problem	45,712	1,260,888	3.9%	120h
	Medication	49,598	961,103	5.3%	100h
	Test	5,091	495,334	4.2%	50h
Weight alg	Problem	66,780	1,239,820	3.2%	70h
	Medication	101,025	909,676	3.1%	60h
	Test	15,032	485,393	2.2%	30h

Weight alg means the combined algorithm.

**Table 4:** Performance from BaiduBaike, baseline and Weight algorithm in the I2B2 corpus (%).

		categories	*p*	*R_O_*	*R_S_*	*R_H_*	*F_O_*	*F_S_*	*F_H_*
i2b2 corpus (English)	Seeds	Problem	100	34.7	15.8	45.5	51.5	27.4	62.6
		Medication	100	35.6	27.7	34.7	52.5	43.4	51.5
		Test	100	45.5	17.8	47.5	62.6	30.3	64.4
	Baseline	Problem	92.0	58.4	25.7	61.4	71.5	40.2	73.6
		Medication	98.3	46.5	35.6	46.5	63.2	52.3	63.2
		Test	91.7	58.4	22.8	56.4	71.4	36.5	69.9
	Weight alg.	Problem	89.7	62.4	28.7	66.3	73.6	43.5	76.3
		Medication	88.3	94.1	75.2	95.0	91.1	81.8	91.6
		Test	86.3	67.3	28.7	66.3	75.6	43.1	75.0
		Drugbank	100	77.2	57.4	67.3	87.2	73.0	80.4
		DrugBank+SNOMED	100	80.2	58.4	70.3	89.0	73.8	82.6
i2b2 corpus (translated to Chinese)	Baidubaike	Problem	94.3	24.8	21.0	74.0	39.3	34.4	82.9
		Medication	98.9	62.7	52.0	67.0	76.8	68.2	79.9
		Test	99.1	20.4	14.7	32.3	33.8	25.6	48.7
	Baseline	Problem	95.5	61.4	47.4	90.2	74.7	63.4	92.8
		Medication	98.3	84.3	70.3	80.7	90.8	82.0	88.6
		Test	95.3	55.0	32.3	62.7	69.7	48.2	75.6
	Weight alg.	Problem	94.9	68.4	52.2	91.6	79.5	67.4	93.2
		Medication	94.0	89.7	74.7	82.7	91.8	83.2	88.0
		Test	94.2	60.1	40.2	67.9	73.4	56.4	78.9
Labeled corpus	Baidubaike	Problem	94.3	20.6	12.0	70.9	33.8	21.3	80.9
		Medication	98.9	60.5	41.7	70.1	75.1	58.7	82.0
		Test	99.1	20.2	15.5	52.7	33.6	26.8	68.8
	Baseline	Problem	95.5	62.9	29.6	92.4	75.8	45.2	93.9
		Medication	98.3	84.4	61.8	80.3	90.8	75.9	88.4
		Test	95.3	42.7	29.7	74.7	59.0	45.3	83.8
	Weight alg.	Problem	94.9	68.1	31.1	95.1	79.3	46.8	95.0
		Medication	94.0	95.6	69.2	81.6	94.8	80.0	87.4
		Test	94.2	47.2	34.3	79.5	62.9	50.3	86.2

Weight alg means the combined algorithm.

### Evaluation methods

Medical terms tend to have many surface variants. For example “慢阻性肺炎” and “慢阻” mean the same object “慢性阻塞性肺炎 (chronic obstructive pulmonary disease)” in Chinese, while “COPD” mean the same object “chronic obstructive pulmonary disease.” Such surface variants would be treated by separate software in actual applications. To reflect an actual situation, we introduce three different Recalls. Surface Recall (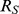) is Recall in the conventional sense (e.g. an exact match of surface forms between terms in text and terms in a collection). The other two (Object Recall (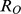), and Surface Head Recall (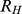)) consider the effect of surface variants by relaxing the matching conditions, as described in the following. Corresponding to these three Recalls, we have three different F-measures (Object F-measure (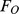), Surface F-measure (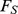), and Surface Head F-measure (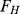)).

One of the foci in this study is how to discard non-terms from a vast number of candidates gathered from the Web. To evaluate the performance of this aspect, we introduce a new metric of error (Err) that measures the false positives among discarded candidates (terms that are discarded by a filtering method).

The followings are definitions of the metrics.

Precision (P): N samples are chosen randomly from a built thesaurus. The number of true entities, M, is obtained by three of the authors after reviewing the samples. The definition of precision is given in Eq. (6). In our task, the N is 1,000.
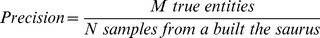
(6)


Object recall (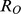): True positive (TP) is defined as the number of objects appearing in both a thesaurus and a reference corpus. They may have different surface expressions. In the previous example, since “慢阻性肺炎” and “慢阻” and “慢性阻塞性肺炎 (chronic obstructive pulmonary disease)” denote the same object, it is counted as a true positive if at least one of these three is registered in the collection.
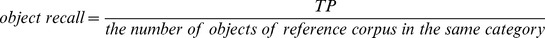
(7)


Surface recall (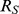): True positive (TP) is defined as the number of strings in a collection that perfectly match those in the reference corpus. “慢阻性肺炎” and “慢阻” are not considered the same expression.
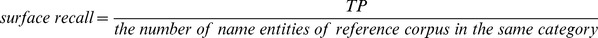
(8)


Surface head recall (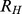): True positive (TP) is defined as the number of head noun strings in the thesaurus perfectly matching those in the reference corpus. The head noun of an entry is obtained by removing all modifiers (e.g. body parts, chronic/acute, etc.) of the central word. Moreover, the relationship between an entry and its head noun should be an “is-a” relationship. For example, the head noun of the entry “脑损伤 (brain lesion)” should be “损伤 (lesion),” removing the body part “脑 (brain).” Another example is the entry “慢性阻塞性肺炎” (chronic obstructive pulmonary disease), whose head noun is “肺炎 (pulmonary disease).” Note that both brain lesion and COPD are special cases of lesion and pulmonary disease.
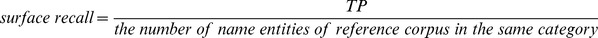
(9)object F-measure (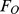):
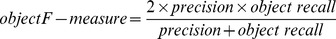
(10)surface F-measure (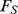):
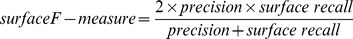
(11)surface head F-measure (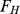):
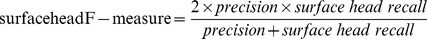
(12)error (true false missing) (Err): N samples are chosen randomly from the removed name entity list. By reviewing the N samples, three of the authors picked up M true entities. The definition of error is given in Equation (13). In our task, the N is 1,000.
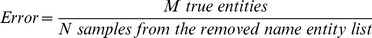
(13)


## Results of Experiments

Two systems were developed, for both English and Chinese. The difference in the two systems is the final phase of removing non-terms. The first one, which we call the baseline system, uses only the snippet analysis. The other system, called the combined system (Weight alg), combines the snippet analysis with the technique of assessing the quality of lists (“parallel” structures) illustrated in the previous section. The latter has proven to be more effective and efficient since it can deal with a term whose frequency on the Web is rather low. We also show results of the seed dictionaries, most of which were generated using Baidu Baike entries and i2b2 labeled named entities, with the exception of the English medication seed dictionary that was generated using SNOMED entries.

Table 3 shows the number of terms collected, the number of candidates removed as non-term, err, and the processing time required (Processor: Intel(R) Core(TM) Quad CPU Q9400 @2.66 GHz 2.67 GHz; RAM: 2.00 GB). As shown in Table 3, both systems successfully removed a large number of non-terms with minimum errors (the err ranges from 2.3 to 5.3% in English).

Table 4 shows the performance in terms of Recall, Precision, and F-measure. The collections of seed terms alone, of terms collected by the baseline system, and of terms collected by the combined system were evaluated. The high precisions imply that both of the filtering methods work well. The lowest precision (86.3%) was observed in the experiment on Test of the English i2b2 by using the collection generated by the combined system. We did not conduct experiments without using our filtering methods because that would introduce a huge amount of noise (on average, our filtering methods removed 80% of entries extracted from the Internet, most of which are irrelevant to target dictionaries) and the noise will also make the iterative algorithm difficult to run. We believe that the precision of our dictionaries will be significantly lower if no filtering method is applied.

However, the two systems have significant differences in efficiency and Recall. The efficiency of the combined system is significantly better than the baseline system. This is because the snippet analysis needs to submit a large number of queries to the search engine. On the other hand, the combined system uses “parallel” structures to extract terms from them at the same time, which reduces the processing time substantially.

As shown in Table 4, the combined method has proven to be the most effective for Medication. Since the English seed terms for Medication are extracted from SNOMED, one can see the coverage of terms in actual medical records by a term collection derived from an existing resource. For comparison, we also include the performances of a collection of Medication derived from Drugbank and a collection generated by using both of SNOMED and DrugBank. The results show that, while DrugBank covers terms much better than SNOMED, the collection of terms constructed by our combined method has a much higher coverage than even the collections that use both SNOMED and Drug.

While the coverages (Recall) and F-measures improve for all three classes, the improvement for Medication is the most significant. That is, while the Surface Recalls by collections from SNOMED and DrugBank are 27.7 and 57.4, the expanded collections by the baseline system and the combined system cover 35.6 and 75.2 of terms in the i2b2 corpus. The combined system significantly outperforms the baseline system. Though the margins aren't so large, the collections by the combined method outperform the baseline systems for Problem and Test as well.

The same tendency in Recall has been observed for Chinese. That is, the combined method consistently outperforms the baseline system and the collections of seed terms alone. As in English, the most significant improvement was observed for Medication, both for the Chinese i2b2 terms and the Chinese labeled corpus. Table 4 also shows that the difference between the objective recall and surface recall (objective recall – surface recall) is bigger than that in i2b2 corpus. However, rather significant differences exist in 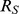, 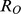, 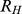 between English and Chinese in certain classes. These differences show some differences in term formation of the two languages, which we will discuss in the next section.

## Discussion

The collection derived from the BaiduBaike has had a rather poor performance, particularly in terms of Recall. This shows that, though a common practice in the general domain, the approach of creating a collection of “important” terms from Web encyclopedias does not work well for term collections for medical record processing. This is because a large number of terms in medical records are not included in an online encyclopedia. For the same reason, another common approach, the snippet analysis, is less effective in the medical domain than in the general domain. The recall of the snippet analysis tends to be rather low, because names of rare Medications, Problems, and Tests do not appear often enough for the snippets analyses to work.

Although our proposed methods need a seed dictionary to search the Internet and crawl related webpages, we believe that the size of the seed dictionary does not have much impact on the final performance of term collection, as long as the precision of the seed dictionary is high enough (i.e. no irrelevant entries in the seed dictionary). This is primarily because of the fact that our algorithm is an iterative process and once an entry is included, it will serve as a seed entry in the next iteration. Particularly, in the case of medication names, the experimental results (Table 4) show that our proposed methods (both baseline and the weighted algorithm) have a better or at least a comparable performance compared to DrugBank, which means a large number of entries in DrugBank have already been included in the expanded dictionary and will serve as seed entries for follow-up iterations. Hence, there is no need to repeat the experiment using DrugBank as the seed dictionary.

In order to deal with the difficulties specific to the medical domain, we have proposed a method that exploits the “parallel” structures on the web pages. The method is more efficient than the snippet analysis and our experiments show that it improves recall significantly.

We have also introduced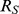,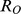, and 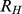 as three different ways to evaluate the recall of a system. 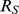 is the standard recall, while the other two are measures to treat the term variability and productivity that are pervasive problems in the medical domain. Experiments show that 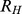 is much higher than 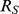 and 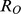. This fact reflects the high productivity shown in medical terms. The term formation of body + problem and body + test (i.e. “脑部 MRI” (brain MRI)) account for a great portion in the Chinese corpus and the labeled corpus. However, some compound terms are not found in webpages crawled by our search engine. “L1 椎体骨折” (L1 vertebral fracture) and “尾骨骨折”(coccyx fracture) are such examples. While 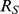 treats them as false negatives, 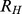
treats them as true positive since they share the same head, e.g. “骨折” (bone fracture). The high productivity of medical terms implies that we cannot enumerate all terms to be created by term formation patterns, and that we have to have dynamic processes (e.g. programs) for accessing dictionaries that embody term formation rules.

The relationship between the head recall and the object recall for English and Chinese collections shows that the two languages are quite different in terms of term formation. That is, while 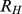 and 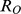 are comparable in English, 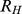 is substantially higher than 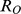 in Chinese. This shows that Chinese medical terms tend to follow term formation patterns more than those in English, partly because of the nature of Chinese characters (ideograms).

Since Chinese characters are ideograms and many Chinese translations of drug names are transliterations, we may have different Chinese translations for the same drug. For instance, “顺泊 (cisplatin)” and “顺铂 (cisplatin)” denote the same medicine and are used by different groups of medical doctors. It is often the case that one translation becomes dominant over others and is found on the web, though other translations continue to be used. This variability caused by translation poses serious challenges, since different translations are used by different hospitals.

There are some differences in the Recall of Medication for the Chinese translation of the i2b2 corpus and the labeled corpus built on Chinese discharge summaries. These differences are caused by different distributions of terms in the two corpora. Because the Chinese labeled corpus is an annotated corpus of actual medical records in China, terms of traditional Chinese medicine frequently occur. Those terms also have high frequency in webpages in Chinese. On the other hand, these terms do not appear in the Chinese translation of the i2b2 corpus because the i2b2 corpus was built on American discharge summaries. In addition, some drugs (especially new ones) used in American hospitals are rarely used in China and may not even have a Chinese translation. These drug names are also typically hard to extract from the Internet. Both factors contribute to the decrease of recall for the Chinese collection of medical terms.

## Conclusion

In this study, our objectives were to develop a language-independent method of building a large collection of medical terms and to use the method to build term collections of Chinese and English for given semantic classes. The method has successfully built collections of terms of high quality and broad coverage. The method requires minimum human intervention. In order to construct a multi-lingual thesaurus, there are still many tasks to perform. These are (1) gathering variants of the same terms, (2) relating terms with richer semantic ontology such as MeSH, etc. and (3) aligning terms in different languages. The present work has established the first step to this direction by constructing large collections of terms in given languages.
