# Correction: Building Large Collections of Chinese and English Medical Terms from Semi-Structured and Encyclopedia Websites

**DOI:** 10.1371/annotation/fab3bd89-3ae3-445f-a418-207bbd7d4377

**Published:** 2013-12-16

**Authors:** Yan Xu, Yining Wang, Jian-Tao Sun, Jianwen Zhang, Junichi Tsujii, Eric Chang

There are multiple symbols missing in the fourth and sixth paragraphs of the Discussion section.

Please see the corrected fourth paragraph here:

In order to deal with the difficulties specific to the medical domain, we have proposed a method that exploits the “parallel” structures on the web pages. The method is more efficient than the snippet analysis and our experiments show that it improves recall significantly.

We have also introduced , , and as three different ways to evaluate the recall of a system. is the standard recall, while the other two are measures to treat the term variability and productivity that are pervasive problems in the medical domain. Experiments show that is much higher than and . This fact reflects the high productivity shown in medical terms. The term formation of body + problem and body + test (i.e. “脑部MRI” (brain MRI)) account for a great portion in the Chinese corpus and the labeled corpus. However, some compound terms are not found in webpages crawled by our search engine. “L1椎体骨折” (L1 vertebral fracture) and “尾骨骨折”(coccyx fracture) are such examples. While treats them as false negatives, treats them as true positive since they share the same head, e.g. “骨折” (bone fracture). The high productivity of medical terms implies that we cannot enumerate all terms to be created by term formation patterns, and that we have to have dynamic processes (e.g. programs) for accessing dictionaries that embody term formation rules.

The sixth paragraph should read:

Since Chinese characters are ideograms and many Chinese translations of drug names are transliterations, we may have different Chinese translations for the same drug. For instance, “顺泊 (cisplatin)” and “顺铂 (cisplatin)” denote the same medicine and are used by different groups of medical doctors. It is often the case that one translation becomes dominant over others and is found on the web, though other translations continue to be used. This variability caused by translation poses serious challenges, since different translations are used by different hospitals.

